# Maternal wellbeing of Malaysian mothers after the birth of a preterm infant

**DOI:** 10.1186/s12884-023-05823-y

**Published:** 2023-07-13

**Authors:** Liz Jones, Jeevitha Mariapun, Abbey Xiao Qian Tan, Zaid Kassim, Tin Tin Su

**Affiliations:** 1 Jeffrey Cheah School of Medicine and Health Sciences, Monash University (Malaysia), Jalan Lagoon Selatan, Bandar Sunway, Selangor Malaysia; 2 2Segamat District Public Health Office, Ministry of Health, 85000 Segamat, Johor Malaysia; 3 South East Asia Community Observatory (SEACO), Monash University (Malaysia), Jalan Lagoon, Selatan, Bandar Sunway, Selangor State Malaysia

**Keywords:** Maternal wellbeing, Preterm infant, Social support, Malaysia

## Abstract

**Background:**

In Malaysia approximately 7% of births result in a preterm birth (< 37 weeks). Research in many other countries has found that mothers of preterm infants experience poorer psychological wellbeing. However, there has been limited research in Malaysia. We examined wellbeing, using the WHO Quality of Life brief version questionnaire (WHOQOL-BREF), in mothers who have preterm and full-term infants.

**Methods:**

Data was collected as part of the South East Asian Community Observatory MISS-P project. A total of 3221 mothers (7.9% with a preterm and 92.1 with a full-term birth) completed a survey, with a range of measures, including the WHOQoL-BREF and sociodemographic questions.

**Results:**

For the physical health, psychological wellbeing and quality of their environment WHOQOL-BREF domains, a lower gestational age, a lower education level, and having had an emergency caesarean delivery were significantly associated (*p* < 0.05) with a lower quality of life, and there was a weak effect for ethnicity for some domains. The effects were strongest for mothers’ education level.

**Conclusions:**

There is a weak but significant relationship between the gestational age of an infant and the mother’s quality of life. Mothers in Malaysia with a preterm infant or a lower level of education may benefit from additional support.

**Supplementary Information:**

The online version contains supplementary material available at 10.1186/s12884-023-05823-y.

In 2020, 6.63% of births in Malaysia were preterm, with the highest rates for Orang Asli and Indian women and women aged 40 years and above [1]. Of preterm births in Malaysia, studies have found between 14.1% and 20.2% were very or extremely preterm [1, 2]. The birth of a preterm infant is highly stressful [3, 4], particularly for parents whose infant has a longer hospital stay [5], with parents describing it as “the worst major life event they have experienced” ([6] p.186). The strain experienced by parents of preterm infants is significantly higher than those of full-term infants [4, 7], is contrary to parents’ expectations regarding birth [6], and requires parents to cope with the stress of their infant’s medical condition and the technologically intensive clinical environment [8]. Most studies find that mothers of preterm infants experience higher levels of stress, and anxiety than mothers of full-term infants [9–11], and some find higher depression [11, 12], but not all [13, 14]. There is also some evidence of higher anxiety and stress for parents of very preterm infants compared to moderately preterm infants [15, 16], but not consistently [11, 17]. Longitudinal research has found after the birth of a very preterm infant, mothers may evidence higher distress [18], and, seven years later, poorer psychological wellbeing and family functioning [19]. In contrast, other studies find no differences longer term in psychological distress for parents of preterm infants [20–22].

However, research to date has focused on Western countries, with limited research into the psychological wellbeing of mothers of preterm infants in Malaysia specifically, and Asia more generally. Differences in health systems, and cultural norms around family relations and support for new mothers, mean we cannot assume findings from Western cultures apply to Malaysia or other Asian countries [23, 24]. Research to date in Malaysia has focused on psychological wellbeing in specific populations, such as mothers with gestational diabetes, where a preterm birth was associated with depression and anxiety [25], or has examined antenatal depression as a predictor of preterm birth [26]. The only study examining a general population sample found mothers of preterm infants experienced high stress and anxiety [24], but there was no comparison with mothers of full-term infants, to identify whether a preterm infant increases a mother’s risk of psychological distress. Chang et al. [15], in a study of mothers in Taiwan, found that 25% of mothers of preterm infants exhibited significant distress 6–48 months after the birth of their infant, with higher rates of distress for mothers of very preterm infants, but again there was no comparison with mothers of full-term infants. Our study examined mothers of both preterm and full-term infants in Malaysia post discharge and up to four months after the birth of their infant.

Moreover, while there has been extensive research on psychological distress in mothers of preterm infants, less research has focused on other measures of wellbeing, such as post-traumatic growth or life satisfaction. Porat-Zyman et al.’s [27] longitudinal study of Israeli mothers of full-term and preterm infants found that while mothers of preterm infants initially experienced more psychological distress, after four years, mothers of preterm infants reported higher personal growth than mothers of term infants. Our study assessed quality of life, which provides a broader assessment of maternal functioning. Our research question was “what is the relationship between gestational age and quality of life in Malaysian mothers following the birth of their infant?

There has also been inconsistency in findings regarding whether maternal demographic characteristics predict psychological wellbeing in mothers of preterm infants, with a call for further research to examine their potential role, including in Malaysia [24]. Appropriate use of resources requires identification of at-risk populations, to ensure interventions are targeted. Thus, our study also examined the relationship between a range of sociodemographic factors and quality of life.

## Method

### Participants and procedure

The study was conducted in the Segamat district where the South East Asian Community Observatory (SEACO) operates. There were 3221 participants in the study recruited between 2013 to 2021. Two hundred and fifty-four had a preterm birth (< 37 weeks gestation) and 2967 had a full-term birth. The Mothers and Infants in SEACO Segamat Project (MISS-P) was established in 2013 to investigate maternal and child health and well-being. All women in five designated sub-districts of SEACO’s catchment area, Bekok, Chaah, Jabi, Gemereh, and Sg Segamat Malaysia, who were known to have given birth were approached by trained data collectors within 0–16 weeks after childbirth. We obtained the monthly birth lists containing basic demographic details of mothers from government clinics. Mothers were approached at their houses by the field team and asked to participate in a structured survey-based interview. Mothers who provided informed consent were interviewed individually, for about 20–40 min. If it was inconvenient to be interviewed, mothers were asked to fill in a questionnaire consisting of the same questions as the interview protocol. Upon the emergence of the COVID-19 pandemic data was collected through telephone interviews. A baby pack was given to each mother as a token of appreciation regardless of whether they consented to an interview. This study was approved by the Monash University Human Research Ethics Committee (Project ID: 19,451).

## Materials

### Demographics

Participants were asked to provide demographic information, including their age, nationality, ethnicity, education level, and marital status. Additionally, participants were asked about their number of pregnancies and infant births.

### Quality of life

Quality of life was measured with the brief, 26-item version of the WHOQOL, which was developed by the World Health Organization [28]. The first two items are single-item measures of an individual’s perceived quality of life and self-reported health satisfaction. The other 24 items are grouped into four domains: physical health (seven items), psychological (six items), social relationship (three items), and environment (eight items). All items were rated on a 5-point scale as appropriate to the item, for example, ranging from 1 (*very poor*) to 5 (*very good*). There are multiple parameters assessed under each domain. Details of this are available in the Supplementary file (Table S1). We used the Malay version of the WHOQO, which has been validated [29].

Domain scores were computed by averaging the items within each domain, with items 3, 4 and 26 reverse-coded. Scores were then transformed to a range between 4–20 (i.e., multiplied by 4), to be comparable with the scores used in the original WHOQOL-100. Higher domain scores indicated a higher perceived quality of life in each respective domain. In this study, the internal consistency of the WHOQOL was marginal for the physical health (*α* = 0.67) domain and acceptable for the psychological (*α* = 0.73), social relationships (*α* = 0.80) and environment (*α* = 0.83) domains. These values are comparable to the internal consistencies of domains reported in the original international field trial of the WHOQOL, which revealed Cronbach’s alpha values ranging from 0.66 to 0.80.

### Infant and birth information

Information regarding the sex of infant (male/female), gestational age (weeks), birth weight (kg), type of delivery (e.g., standard/breech vaginal delivery), birth complications (if any), and APGAR scores (at 1 and 5 min) were obtained from the baby record books available at the clinics in the relevant sub-district. Maternal breastfeeding habits were also assessed, including duration and reasons for not breastfeeding. Approximately 7.9% of births were preterm (< 37 weeks gestation), while 92.1% were full-term births. Only 0.5% of births (*N* = 15) were very preterm.

### Perceived social support

Perceived social support was assessed with two items, in which mothers reported the type of support they received since their infant’s birth (e.g., no support, assistance if sick, someone to talk to) and who their primary supporter was (e.g., husband, mother, father, extended family).

## Statistical analyses

All variables had less than 2.5% of missing data, with the exception of the variables ‘smoking’ (missing = 44.64%), ‘intake of regular medication during pregnancy’ (missing = 44.92%) and ‘reasons for not breastfeeding’ (missing = 11.29%) which were not considered for the regression analyses. For the WHOQOL-BREF domains, any necessary cleaning was carried out in accordance with the official guideline provided. If any two items were missing from the domain, the domain score was not calculated. This is with the exception of Domain 3, where the domain score was calculated if less than 1 item was missing. The domain scores were also transformed to a range between 4–20, to be comparable with the WHOQOL-100.

Normality tests were carried out for all continuous variables being analysed. For the regression analysis, the *p* values for the F-test for all WHOQOL-BREF domains were statistically significant (> 0.0001) and the R^2^ ranged from 0.02 to 0.07. Multicollinearity tests were carried out using variance inflation factor. The assumption of linearity and the presence of influential points and outliers were checked. Besides that, the test for goodness of fit, independence of observations, homoscedasticity of residuals, and model specification were also considered. Further details on the data cleaning, recoding, missing data (Tables S2-4).

Descriptive analyses were carried out on the relevant sociodemographic and clinical characteristics of the mothers and infants, the breastfeeding habits of the mother and the WHOQOL-BREF domains comparing mothers of preterm and full-term infants. When comparing the values between the two gestational categories, chi-square test was used for the categorical variables, and if the assumptions for the chi-square test were not met, Fisher’s exact test was utilised. For the continuous variables, the Wilcoxon rank-sum test was utilised.

Then, simple regression analysis, as well as multiple regression analysis, were performed, which enabled us to test more accurately the relative effect of infant gestational age. The association between the sociodemographic and clinical variables and each of the four WHOQOL-BREF domains was examined, with the scores of the domains acting as the dependent variable. A p value < 0.05 was considered statistically significant. All statistical analyses were carried out using Stata version 17 (StataCorp LLC, College Station, TX).

## Results

### Descriptive results

The sociodemographic and clinical characteristics of the mothers are presented in Table 1. The median age of the mothers in the full-term group was 29 years (IQR 26–33), and in the preterm group was 30 years (IQR 26–34). Most participants were Malaysian for both full-term (94.0%) and preterm (96.8%) groups, with the vast majority being of Malay ethnicity for both full-term (74.7%) and preterm (72.3%), followed by Chinese, Indian, and indigenous groups and others. In terms of education level, only 27.2% of mothers who had preterm births and 28.2% who had full-term births had completed a pre-university or university education. The majority of mothers were married for both full-term (98.3%) and preterm (96.0%) groups, and all mothers in the study were the principal carer of their infant. In terms of social support, most mothers had someone they could rely on if they needed help (personal and infant health), and their main primary support was their husband for both full-term (93.9%) and preterm (89.2%) groups. The only differences between mothers of preterm and full-term infants were that mothers of preterm infants were slightly less likely to be married.

**Table 1:** Maternal sociodemographic and clinical characteristics by gestational categories, *n* = 3221

Variables	n	Preterm (< 37 weeks), *n* = 254	Full-Term (37 or more weeks), *n* = 2967	*p value*
Age of Mother, years, Median (IQR)	3213	30 (26–34)	29 (26–33)	0.456
Nationality	3221			0.060
Malaysian		245 (96.8)	2783 (94.0)	
Non-Malaysian		8 (3.2)	179 (6.0)	
Ethnicity^a^	3216			0.183
Malay		183 (72.3)	2212 (74.7)	
Chinese		33 (13.0)	395 (13.3)	
Indian		16 (6.3)	184 (6.2)	
Other Bumiputera^b^		17(6.7)	108 (3.6)	
Others^c^		4 (1.6)	64 (2.2)	
Highest Education Level	3211			0.972
Never attended school, Primary		23 (9.09)	258 (8.7)	
Secondary		161 (63.6)	1867 (63.1)	
College (Pre-University)		34 (13.4)	429 (14.5)	
University		35 (13.8)	404 (13.7)	
Marital Status	3211			0.023*
Married		242 (96.0)	2910 (98.3)	
Single, Separated/Living Apart, Divorced, Widowed		10 (4.0)	49 (1.7)	
Number of infants born^d^ (Recent births), Median (IQR)	3221	1 (1–1)	1 (1–1)	< 0.001**
Took regular medication during pregnancy	1774			0.331
Yes		129 (77.7)	1300 (80.9)	
No		37 (22.3)	308 (19.2)	
Smoking during pregnancy	1783			0.773
Yes		4 (2.4)	33 (2.0)	
No		164 (97.6)	1582 (98.0)	
Mother the principal carer	3221			-
Yes		239 (100)	2967 (100)	
No		-	-	
Type of support after birth	3205			0.019*
No support, sole carer		8 (3.2)	63 (2.1)	
Someone to help with baby-care, if I were sick		193 (76.6)	2254 (76.3)	
Someone to take me or the baby a clinic or hospital if needed		162 (64.3)	1658 (56.2)	
Someone to talk to about any problems		92 (36.5)	985 (33.4)	
Financial support		51 (20.2)	522 (17.7)	
Primary support	3199			0.040*
Husband		223 (89.2)	2754 (93.9)	
Parents		22 (8.8)	153 (5.2)	
Mother-in-law		3 (1.2)	15 (0.5)	
Extended family/Friend, Paid help, Others		2 (0.8)	27 (0.9)	

Values are numbers (percentage) unless stated otherwise

^a^Percentage was calculated from a total that excluded the “missing” & “refused to answer” categories

^b^Other Bumiputeras are the indigenous groups of Peninsular Malaysia (the Orang Asli) and East Malaysia

^c^Others includes all other non-native Malaysian citizens

^d^Number of infants born (recent births) refers to the median number of births in the recent delivery (98.9% of mothers had singleton births);

^*^Statistically significant (*p* < 0.05); ** Statistically significant (*p* < 0.001)

The sociodemographic and clinical characteristics of the infants and the breastfeeding habits of the mother are presented in Table 2. There was a significant weight difference (*p* < 0.001) between the 2 gestational groups, with the median weight for full-term infants being 3.1 kg (IQR 2.8–3.4) and the preterm group being 2.4 kg (IQR 2.0–2.9). Most infants were born through the standard/breech vaginal delivery for both full-term (77.8%) and preterm (65.0%) categories, however there were significantly higher cases of emergency caesarean for the preterm group (27.2%) compared to the full-term group (12.9%). Most infants were also found to have no birth complications for both full-term (81.1%) and preterm (70.5%), and this was reflected in the APGAR scores (at one minute and at five minutes), with the median score being 9 for both groups. In terms of breastfeeding habits, most mothers did breastfeed their infants for both the full-term (98.3%) and preterm (96.1%) groups, with the median duration of the breastfeeding being 11 weeks (IQR 7–17) for full-term and 12 weeks (IQR 7–17) for preterm at the time of the survey.

**Table 2:** Infants’ sociodemographic and clinical characteristics and maternal breastfeeding habits by gestational categories, *n* = 3221

Variables	n	Preterm (< 37 weeks), *n* = 254	Full-Term (37 or more weeks), *n* = 2967	*p value*
**Infant Characteristics**				
Sex of infant	3221			0.361
Male		127 (50.0)	1572 (53.0)	
Female		127 (50.0)	1395 (47.0)	
Gestational age, Median (IQR)	3221	36 (35–36)	39 (38–39)	< 0.001**
Infant’s birth weight (kg), Median (IQR)	3221	2.4 (2.0–2.9)	3.1 (2.8–3.4)	< 0.001**
Type of delivery	3219			< 0.001**
Standard/Breech Vaginal delivery		165 (65.0)	2306 (77.8)	
Forceps/ Vacuum vaginal delivery		3 (1.2)	77 (2.6)	
Elective Caesarean		17 (6.7)	200 (6.8)	
Emergency Caesarean		69 (27.2)	382 (12.9)	
Type of birth complications	3177			< 0.001**
None		179 (70.5)	2405 (81.1)	
Foetal distress		17 (6.7)	172 (5.8)	
Prolonged labour		10 (3.9)	86 (2.9)	
Meconium stained liquor		3 (1.2)	39 (1.3)	
Neonatal jaundice		33 (13.0)	243 (8.2)	
Congenital abnormalities		-	4 (0.1)	
Maternal postpartum haemorrhage		10 (3.9)	24 (0.8)	
APGAR score (1 min), Median (IQR)	3152	9 (9–9)	9 (9–9)	< 0.001**
APGAR score (5 min), Median (IQR)	3153	9 (9–9)	9 (9–9)	0.174
**Maternal Breastfeeding Habits**				
Breastfed Infant	3216			0.015*
Yes		243 (96.1)	2911 (98.3)	
No		10 (4.0)	52 (1.8)	
Reasons for not breastfeeding^b^, *n* = 62	55			1.000
Did not want to		3 (42.9)	22 (45.8)	
Wanted to, but was not able to		3 (42.9)	21 (43.8)	
Was not allowed to		1 (14.3)	5 (10.4)	
Duration of breastfeeding (weeks)^c^, Median (IQR), *n* = 3154	3126	12 (7–17)	11(7–17)	0.975

Values are numbers (percentage) unless stated otherwise

^*^Statistically significant (*p* < 0.05); ** Statistically significant (*p* < 0.001)

^a^Percentage was calculated from a total that excluded the “missing” & “refused to answer” categories

^b^Only includes mothers who have never breastfed their babies

^c^Only includes mothers who are still breastfeeding until the point of the interview

^d^Only includes mothers who have breastfed but are no longer breastfeeding

### Main findings

The results of the comparisons between mothers of preterm and full-term infants are presented in Table S5. The maternal and infant factors associated with the WHOQOL-BREF physical health, psychological health, social relationships, and environmental health domains are shown in the simple linear regression models (see Table S6). Variables that were significant in the simple linear regression analysis or known clinical and social predictors for each WHOQOL-BREF domain were included in the multiple linear regression model (see Table 3)^1^. The multiple linear regression analyses show that for most of the WHOQOL-BREF domains, a lower gestational age, a lower education level, and having had an emergency caesarean delivery were significantly associated (*p* < 0.05) with a lower quality of life.

**Table 3:** Associations of quality of life domains with maternal and infant sociodemographic and clinical characteristics (multiple linear regression)

Variable	Domain 1 (Physical health), *n* = 3021	*p* value	Domain 2 (Psychological), *n* = 3020	*p* value	Domain 3 (Social Relationship), *n* = 2973	*p* value	Domain 4 (Environment), *n* = 3020	*p* value
Gestational age (weeks)	0.0508 (0.009–0.093)	0.018*	0.044 (0.003–0.085)	0.035*	0.011 (-0.037–0.058)	0.660	0.079 (0.027–0.130)	0.003*
Nationality								
Malaysian (ref)								
Non-Malaysian	-0.298 (-0.592 – -0.005)	0.046*	-0.293 (-0.558- -0.028)	0.030*	-0.204 (-0.490- 0.082)	0.162	-0.231 (-0.582- 0.119)	0.195
Ethnicity								
Malay (ref)								
Chinese	0.0670 (-0.123–0.256)	0.488	-	-	-0.263 (-0.469- -0.056)	0.013*	0.053 (-0.169–0.276)	0.639
Indian	-0.191(-0.423–0.040)	0.105	-	-	-0.005 (-0.276–0.287)	0.970	0.367 (0.082–0.651)	0.012*
Other Bumiputera	0.922 (0.619–1.225)	< 0.001**	-	-	-0.291 (-0.574- -0.008)	0.044*	-0.946 (-1.278- -0.613)	< 0.001**
Others	0.200 (-0.334–0.733)	0.463	-	-	-0.262 (-0.759- 0.235)	0.301	-0.253 (-0.818- 0.313)	0.381
Highest Education Level								
University (ref)								
Never attended school, Primary	-0.413 (-0.710- -0.116)	0.006*	-0.568 (-0.828- -0.308)	< 0.001**	-0.298 (-0.599- 0.004)	0.053	-1.214 (-1.523- -0.905)	< 0.001**
Secondary	-0.206 (-0.409- -0.003)	0.047*	-0.424 (-0.601- -0.247)	< 0.001**	-0.262 (-0.460- -0.063)	0.010*	-0.705 (-0.891- -0.518)	< 0.001**
College (Pre-University)	-0.048 (-0.301–0.206)	0.712	-0.292 (-0.517- -0.068)	0.011*	0.043 (-0.202- 0.287)	0.732	-0.185 (-0.418- 0.049)	0.121
Marital Status								
Married (ref)								
Single, Separated/Living Apart, Divorced, Widowed	-	-	-0.151 (-0.748- 0.446)	0.620	-0.221 (-0.826- 0.384)	0.473	-0.084 (-0.630- 0.461)	0.763
Primary support								
Husband (ref)								
Parents	-	-	-0.436 (-0.761- -0.111)	0.009*	-0.374 (-0.755- 0.006)	0.054	-0.195 (-0.566- 0.177)	0.304
Mother-in-law	-	-	-0.786 (-1.575- 0.004)	0.051	-1.346 (-2.339- -0.354)	0.008*	-0.541 (-1.282–0.200)	0.152
Extended family/Friend, Paid help, Others	-	-	-0.854 (-1.800- 0.092)	0.077	-0.726 (-1.845- 0.393)	0.203	-0.707 (-1.566- 0.152)	0.107
Sex of infant								
Male (ref)								
Female	-	-	-	-	-0.114 (-0.245–0.016)	0.086	-	-
Infant’s birth weight (kg)	-	-	-	-	-	-	-0.113 (-0.278–0.051)	0.177
Type of delivery								
Standard Vaginal/ Breech delivery (ref)								
Forceps/Vaccum vaginal delivery	0.229 (-0.206- 0.663)	0.302	-0.046 (-0.443- 0.352)	0.822	-0.019 (-0.466- 0.428)	0.933	0.021 (-0.406- 0.449)	0.923
Elective Caesarean	0.047 (-0.211–0.305)	0.723	-0.023 (-0.288- 0.241)	0.862	-0.095 (-0.377–0.187)	0.509	0.223 (-0.047–0.492)	0.105
Emergency Caesarean	-0.252 (-0.442- -0.063)	0.009*	-0.305 (-0.489- -0.122)	0.001**	-0.197 (-0.395- -0.001)	0.051	-0.316 (-0.509- -0.124)	0.001**
APGAR score (1 min)	0.095 (0.039–0.151)	0.001*	0.054 (0.002–0.106)	0.040*	-	-	0.010 (-0.048–0.068)	0.730

Values presented are the unstandardised beta coefficients (B) and corresponding 95% CIs; ^a^All mothers answered ‘Yes’ for this variable; *Statistically significant (*p* ≤ 0.05); **Statistically significant (*p* ≤ 0.001)

In the physical health domain, a higher gestational age, those of indigenous ethnicity versus Malays, and those with a higher one-minute Apgar score were associated with a higher quality of life, while non-citizens, those with less than a pre-university education compared to those with a university education, and those who had an emergency caesarean versus a standard delivery had a lower quality of life. For the psychological health domain, a higher gestational age, and a higher one-minute Apgar score were associated with a higher quality of life, while non-citizens, those with less than a university education, those who were primarily supported by their parents versus their husbands, and those who had an emergency caesarean had a lower quality of life.

For the social relationships domain those of indigenous or Chinese ethnicity, and those who were primarily supported by their mothers-in-law had a lower quality of life. In terms of education level, when compared to those with a university education, those with lower than a pre-university education had negative associations with quality of life but only the secondary education category was statistically significant. In the environmental health domain, a higher gestational age, and those of Indian ethnicity, were associated with a higher quality of life, while those of indigenous ethnicity, those with less than a pre-university education versus university education, and those who had an emergency caesarean had a lower quality of life.

## Discussion

Our study examined the relationship between a preterm birth and Malaysian mothers’ quality of life across the 4 domains of the QoL scale. As a secondary aim we investigated whether there was a relationship between demographic factors and QoL. Overall, we found that there was a positive relationship between gestational age of the infant and mothers’ self-reported physical health, psychological wellbeing and quality of their environment. No relationship was found for the domain of social relationships. Our findings for psychological wellbeing are consistent with much previous research finding that the birth of a preterm infant is associated with higher rates of stress, depression and anxiety [9–12, 15, 16].

It is not surprising that poorer physical health was associated with lower gestational age, given the higher caesarean rate in mothers of preterm infants, as well as the association between a range of medical conditions and a higher rate of preterm birth. However, it is notable that these differences were still evident some weeks after the birth of the infant. It is less obvious why there is a significant relationship between gestational age and satisfaction with the quality of their environment. Follow up research is needed to explore this finding.

At the same time, it is important not to overstate our findings, as we were only able to explain a relatively small proportion of the variances in mothers’ scores for the four domains. The effects were found when we used gestation age as a continuous variable rather than as a categorical variable, which suggests that the effects are stronger for mothers of more preterm infants. However, we did not have sufficient sample size to test between groups of preterm and very preterm infants. In addition, our findings demonstrated that the strongest effects were for education, as well as some effects for ethnicity. A number of previous studies across varied countries including Spain, Portugal, and Iran have similarly found effects for education on QoL [30–32].

Our findings demonstrate that mothers of preterm infants in Malaysia, as well as mothers with lower levels of education or of a minority ethnic group, may benefit from interventions targeting their physical and psychological health following the birth of a preterm infant. While there is evidence regarding the effectiveness of interventions in a range of countries [33, 34] to date, these interventions have not been trialed in Malaysia, where there are unique cultural values that reflect the multiple racial groups and languages of Malaysians. Consideration also needs to be given to support the needs of mothers with a lower education level.

There are a number of limitations to our study. Our study was conducted in one region in Malaysia that has a higher proportion of Malays and other Bumiputera (indigenous peoples) and lower levels of income and education than the overall population. Moreover, mothers were surveyed within the first 16 weeks of their infant’s life. Future research needs to examine whether the differences between mothers of term and preterm infants dissipate over time, as found in some other studies.

In conclusion, much past research has focused on psychological distress in mothers of preterm infants, predominantly in western, educated, industrialised, rich democratic (WEIRD) countries. While psychological distress may be important for diagnostic purposes, there is an increasing recognition of the importance of considering other measures of wellbeing. Our study examined the life satisfaction of mothers who had term and preterm infants in Malaysia, finding that gestational age and education had a positive relationship with a number of domains of life satisfaction.

## Supplementary Information

**Additional file 1.** Supplementary materials.docx. Information on data cleaning and recoding, and results from additional analyses. 

## Data Availability

Available upon request from first author. However, permission must also be given by SEACO for any sharing of their data.

## Footnotes

1 The results for the multiple linear regression analyses using infant status as a categorical variable are presented in Table S6.
