# Supplementary material for: Maternal wellbeing of Malaysian mothers after the birth of a preterm infant
Source: BMC Pregnancy Childbirth. 2023 Jul 13;23:510. doi: 10.1186/s12884-023-05823-y (PMC10339622; doi:10.1186/s12884-023-05823-y)
Supplement: Supplementary file 1 — Additional file 1. Supplementary materials.docx. Information on data cleaning and recoding, and results from additional analyses. [file 12884_2023_5823_MOESM1_ESM.docx]

**SUPPLEMENTARY INFORMATION**

**Data Cleaning and Recoding**

Before the main analyses were carried out, the data was cleaned and a few variables were recoded or categorised. In regards to missing data, all variables had less than 2.5% of missing data with the exception of the variables ‘smoking’ (missing = 44.64%), ‘intake of regular medication during pregnancy’ (missing = 44.92%) and ‘reasons for not breastfeeding’ (missing =11.29%) which were not considered for the regression analyses. Besides that, any data that didn’t make logical sense, fell out of a sensible range or had responses recorded as ‘refused to answer’, were recoded as missing. After data cleaning, a total of 3221 mother and infant pairs were included for the statistical analyses.

For the ‘duration of breastfeeding’ variable, additional calculations were carried out as the item that originally measured breastfeeding duration only included mothers who no longer breastfed at the time of the interview. Therefore, in order to calculate the duration of breastfeeding for mothers who were still breastfeeding at the time of interview, the date of interview was subtracted from the date of birth of the infant. This new variable was created under the assumption that the mothers were breastfeeding from the time the infant was born. Besides that, a few other variables were recategorised for a more accurate analysis. For ‘Nationality’, any mothers that identified as foreigners were grouped under the category ‘Non-malaysians’; as they consist of less than 10% of the total sample. For ‘Type of Delivery’, standard and breech vaginal delivery were grouped into one category, while forceps and vacuum vaginal delivery were grouped into another category; as they both share similar delivery characteristics. For the ‘Marital Status’ variable, all the non-married groups were categorised into one group. This includes those who are single, separated, divorced and widowed. This is because married mothers consist of the vast majority, with around 96-98% of the sample being married mothers. For the ‘Primary Support’ variable, extended family, paid help, friend and none of the above were grouped under the category, ‘Others’ as the combined four categories make up for less than 1% of the sample.

For the WHOQOL-BREF domains, any necessary cleaning was carried out in accordance with the official guideline provided. If any two items were missing from the domain, the domain score was not calculated. This is with the exception of Domain 3, where the domain score was calculated if less than 1 item was missing. The domain scores were also transformed to a range between 4-20, to be comparable with the WHOQOL-100. However, the scores for WHOQOL-1 and WHOQOL-2 were left as they are between a range of 1-5.

Normality tests were carried out for all continuous variables being analysed. For the regression analysis, the *p* values for the F-test for all WHOQOL-BREF domains were statistically significant (> 0.0001) and the R^2^ ranged from .02 to .07. Multicollinearity tests were carried out using variance inflation factor. The assumption of linearity and the presence of influential points and outliers were checked. Besides that, the test for goodness of fit, independence of observations, homoscedasticity of residuals, and model specification were also considered.

**Table S1.** WHO-BREF Items and Scoring

| Questionnaire Items | Scaling Direction | Item Score Range | Domain Score Range |
| --- | --- | --- | --- |
| How would you rate your quality of life? | Positive | 1-5 |  |
| How satisfied are you with your health? | Positive | 1-5 |  |
| **Domain 1: Physical Health** |  |  | 7-35 |
| To what extent do you feel that physical pain prevents you from doing what you need to do? | Negative | 1-5 |  |
| How much do you need any medical treatment to function in your daily life? | Negative | 1-5 |  |
| Do you have enough energy for everyday life? | Positive | 1-5 |  |
| How well are you able to get around? | Positive | 1-5 |  |
| How satisfied are you with your sleep | Positive | 1-5 |  |
| How satisfied are you with your ability to perform your daily living activities? | Positive | 1-5 |  |
| How satisfied are you with your capacity for work? | Positive | 1-5 |  |
| **Domain 2: Psychological** |  |  | 6-30 |
| How much do you enjoy life? | Positive | 1-5 |  |
| To what extent do you feel your life to be meaningful? | Positive | 1-5 |  |
| How well are you able to concentrate? | Positive | 1-5 |  |
| Are you able to accept your bodily appearance? | Positive | 1-5 |  |
| How satisfied are you with yourself? | Positive | 1-5 |  |
| How often do you have negative feelings such as blue mood, despair, anxiety, depression? | Negative | 1-5 |  |
| **Domain 3: Social Relationships** |  |  | 3-15 |
| How satisfied are you with your personal relationships? | Positive | 1-5 |  |
| How satisfied are you with your sex life? | Positive | 1-5 |  |
| How satisfied are with the support you get from your friends? | Positive | 1-5 |  |
| **Domain 4: Environment** |  |  | 8-40 |
| How safe do you feel in your daily life? | Positive | 1-5 |  |
| How healthy is your physical environment? | Positive | 1-5 |  |
| Have you enough money to meet your needs? | Positive | 1-5 |  |
| How available to you is the information that you need in your daily-to-day life? | Positive | 1-5 |  |
| To what extent do you have the opportunity for leisure activities? | Positive | 1-5 |  |
| How satisfied are you with the condition of your living place? | Positive | 1-5 |  |
| How satisfied are you with your access to health services? | Positive | 1-5 |  |
| How satisfied are you with your transport? | Positive | 1-5 |  |

Note see https://www.google.com/url?q=https://apps.who.int/iris/rest/bitstreams/59977/retrieve&sa=D&source=docs&ust=1682046775703924&usg=AOvVaw23FRmxb5MH6oQW5lY5ufXx for further information

**Table S2.** Total Missing data for Maternal Characteristics, n=3221

| Variables | Missing, n (%) |
| --- | --- |
| Age of Mother, years, Median (IQR) | 8 (0.25) |
| Nationality | 6 (0.19) |
| Ethnicity | 5 (0.16) |
| Highest education level | 10 (0.31) |
| Marital Status | 10 (0.31) |
| Number of infants born (Recent births) | 0 (0.00) |
| Took regular medication during pregnancy | 1447 (44.92) |
| Smoking during pregnancy | 1438 (44.64) |
| Mother the principal carer | 0 (0.00) |
| Type of support after birth | 16 (0.50) |
| Primary support | 22 (0.68) |
| Physical health | 0 (0.00) |
| Psychological health | 1 (0.03) |
| Social relationship | 49 (1.55) |
| Environmental health | 1 (0.03) |

Percentage includes the ‘missing’ and ‘refused to answer categories’.

**Table S3.** Total Missing data for Infants’ Characteristics and Maternal Breastfeeding Habits, n=3221

| Variables | Missing, n (%) |
| --- | --- |
| **Infant Characteristics** |  |
| Sex of infant | 0 (0.00) |
| Gestational age | 0 (0.00) |
| Infant’s birth weight (kg) | 10 (0.31) |
| Type of delivery | 2 (0.06) |
| Type of birth complications | 44 (1.37) |
| APGAR score (1 min) | 69 (2.14) |
| APGAR score (5 min) | 68 (2.11) |
| **Maternal Breastfeeding Habits** |  |
| Breastfed Infant | 5 (0.16) |
| Reasons for not breastfeeding, n =62 | 7 (11.29) |
| Duration of breastfeeding (weeks), n =3154 | 28 (0.89) |

Percentage includes the ‘missing’ and ‘refused to answer categories’.

**Table S4.** Quality of life by gestational categories

| Parameters | n | Preterm  (<37 weeks), n = 254 | Full-Term  (37 or more weeks), n = 2967 | *p* value |
| --- | --- | --- | --- | --- |
|  |  | **Median (IQR)^a^** | **Median (IQR)** |  |
| Domain 1 (Physical health) | 3221 | 16.0 (14.3-17.1) | 16.0 (14.7-17.1) | 0.525 |
| Domain 2 (Psychological) | 3220 | 16.3 (15.3-17.3) | 16.0 (15.3-16.7) | 0.668 |
| Domain 3 (Social Relationship) | 3171 | 16 (16-16) | 16 (16-16) | 0.783 |
| Domain 4 (Environment) | 3220 | 15.5 (13.5-16.5) | 15.5 (14.0-16.0) | 0.799 |

**^a^** Median reported as data was skewed.

*Statistically significant (*p*<0.05); ** Statistically significant (*p* <0.001).

Where more than two items were missing from the domain, the domain score was not calculated (with the exception of Domain 3, where the domain was calculated if < 1 item was missing).

The scores for WHOQOL-1 and WHOQOL-2 are between a range of 1-5.

The domain scores were transformed to a range between 4-20, to be comparable with the WHOQOL-100.

**Table S5.** Simple Linear Regression Models for the WHOQOL-BREF domains

| Variable | Domain 1  (Physical health),  n = 3021 | *p* value | Domain 2 (Psychological),  n =3020 | *p* value | Domain 3  (Social Relationship),  n = 2973 | *p* value | Domain 4 (Environment),  n = 3020 | *p* value |
| --- | --- | --- | --- | --- | --- | --- | --- | --- |
| Gestational age (weeks) | 0.0534 (0.010-0.097) | 0.016* | 0.059 (0.016-0.101) | 0.007* | 0.022 (-0.022-0.065) | 0.336 | 0.086 (0.040-0.132) | <0.001** |
| Gestational Categories |  |  |  |  |  |  |  |  |
| Full-Term (ref) |  |  |  |  |  |  |  |  |
| Preterm | 0.073 (-0.167-0.312) | 0.552 | -0.062 (-0.296- 0.172) | 0.603 | -0.070 (-0.311-0.172) | 0.572 | -0.096 (-0.348-0.157) | 0.456 |
| Age of Mother, years | -0.001 (-0.013-0.011) | 0.861 | 0.002 (-0.009- 0.014) | 0.674 | -0.001 (-0.012-0.011) | 0.917 | 0.006 (-0.007-0.018) | 0.379 |
| Nationality |  |  |  |  |  |  |  |  |
| Malaysian (ref) |  |  |  |  |  |  |  |  |
| Non-Malaysian | -0.391 (-0.668 –  -0.115) | 0.006* | -0.408 (-0.678-  -0.138) | 0.003* | -0.408 (-0.687-  -0.123) | 0.004* | -0.607 (-0.898- -0.316) | <0.001** |
| Ethnicity |  |  |  |  |  |  |  |  |
| Malay (ref) |  |  |  |  |  |  |  |  |
| Chinese | 0.004 (-0.192-0.201) | 0.966 | -0.110 (-0.302- 0.083) | 0.265 | -0.361 (-0.561-  -0.161) | <0.001** | -0.057 (-0.262-0.148) | 0.583 |
| Indian | -0.236 (-0.512-0.029) | 0.081 | 0.128 (-0.132-0.388) | 0.334 | -0.517 (-0.377-0.161) | 0.430 | 0.236 (-0.040-0.513) | 0.094 |
| Other Bumiputera | 0.696 (0.368-1.023) | <0.001** | -0.042 (-0.361-0.278) | 0.799 | -0.517 (-0.845-  -0.188) | 0.002* | -1.535 (-1.875-  -1.194) | <0.001** |
| Others | -0.143 (-0.610-0.325) | 0.550 | -0.450 (-0.908-0.008) | 0.054* | -0.484 (-0.954-  -0.015) | 0.043* | -0.767 (-1.254-  -0.280) | 0.002* |
| Highest Education Level |  |  |  |  |  |  |  |  |
| University (ref) |  |  |  |  |  |  |  |  |
| Never attended school, Primary | -0.239  (-0.515-0.037) | 0.089 | -0.716 (-0.984-  -0.447) | <0.001** | -0.526 (-0.804-  -0.248) | <0.001** | -1.609 (-1.893-  -1.325) | <0.001** |
| Secondary | -0.195  (-0.386- -0.004) | 0.045* | -0.445 (-0.631-  -0.260) | <0.001** | -0.318 (-0.511-  -0.125) | 0.001** | -0.726 (-0.921-  -0.529) | <0.001** |
| College (Pre-University) | -0.035  (-0.277-0.206) | 0.774 | -0.296 (-0.531-  -0.060) | 0.014* | 0.013 (-0.232-  -0.258) | 0.919 | -0.210 (-0.459-  0.039) | 0.098 |
| Marital Status |  |  |  |  |  |  |  |  |
| Married (ref) |  |  |  |  |  |  |  |  |
| Single, Separated/Living Apart, Divorced, Widowed | 0.144 (-0.345-  0.633) | 0.564 | -0.635 (-1.112-  -0.159) | 0.009* | -0.656 (-1.171-  -0.141) | 0.013* | -0.478 (-0.992-  0.037) | 0.069 |
| Number of infants born (Recent births) | -0.298 (-0.804-0.209) | 0.250 | -0.456 (-0.954-0.038) | 0.070 | -0.222 (-0.730-0.287) | 0.393 | -0.551 (-1.085-  -0.018) | 0.043* |
| Primary support |  |  |  |  |  |  |  |  |
| Husband (ref) |  |  |  |  |  |  |  |  |
| Parents | -0.045 (-0.328-0.239) | 0.758 | -0.539 (-0.814- -0.264) | <0.001** | -0.506 (-0.792-  -0.220) | 0.001** | -0.508 (-0.806-  -0.211) | 0.001** |
| Mother-in-law | -0.283 (-1.141-0.576) | 0.519 | -0.965 (-1.800-  -0.131) | 0.023* | -1.550 (-2.408-  -0.692) | <0.001** | -0.676 (-1.578-0.225) | 0.141 |
| Extended family/Friend, Paid help, Others | -0.105 (-0.787-0.578) | 0.764 | -0.952 (-1.616-  -0.289) | 0.005* | -0.883 (-1.606-  -0.160) | 0.017* | -1.005 (-1.722-  -0.289) | 0.006* |
| Sex of infant |  |  |  |  |  |  |  |  |
| Male (ref) |  |  |  |  |  |  |  |  |
| Female | 0.041 (-0.087-0.170) | 0.528 | -0.050 (-0.176- 0.075) | 0.433 | -0.112 (-0.242-0.018) | 0.091 | 0.0009 (-0.135-0.136) | 0.990 |
| Infant’s birth weight (kg) | 0.030 (-0.110-0.170) | 0.671 | 0.030 (-0.107-0.167) | 0.666 | 0.054 (-0.088-0.195) | 0.458 | 0.105 (-0.042-0.253) | 0.162 |
| Type of delivery |  |  |  |  |  |  |  |  |
| Standard Vaginal/ Breech delivery (ref) |  |  |  |  |  |  |  |  |
| Forceps/Vaccum vaginal delivery | 0.231 (-0.188-  0.650) | 0.280 | -0.057 (-0.466-  0.352) | 0.784 | -0.060 (-0.481-  0.361) | 0.780 | 0.029 (-0.411-  0.470) | 0.896 |
| Elective Caesarean | 0.031 (-0.234-0.296) | 0.819 | 0.018 (-0.241- 0.276) | 0.894 | -0.073 (-0.340-0.195) | 0.594 | 0.344 (0.065-0.624) | 0.016* |
| Emergency Caesarean | -0.267 (-0.452-  -0.082) | 0.005* | -0.320 (-0.500-  -0.140) | 0.001** | -0.190 (-0.378-  -0.003) | 0.047* | -0.302 (-0.496-  -0.107) | 0.002* |
| APGAR score (1 min) | 0.105 (0.035-0.176) | 0.003* | 0.081 (0.012-0.150) | 0.022* | 0.024 (-0.047-0.095) | 0.508 | 0.055 (-0.020-0.129) | 0.149 |
| APGAR score (5 min) | 0.132 (0.032-0.231) | 0.009* | 0.080 (-0.017-0.177) | 0.105 | 0.002 (-0.098-0.102) | 0.968 | 0.070 (-0.035-0.175) | 0.189 |
| Duration of breastfeeding (weeks) | -0.00006 (-0.0006- 0.0005) | 0.831 | 0.0001 (-0.0004-0.0007) | 0.665 | 0.000002 (-0.0006 0.0006) | 0.994 | 0.0003 (-0.0003-0.0009) | 0.322 |

Values presented are the unstandardised beta coefficients (B) and corresponding 95% CIs; ^a^All mothers answered ‘Yes’ for this variable; * Statistically significant (*p*≤0.05); ** Statistically significant (*p* ≤0.001).

**Table S6.** Multiple Linear Regression Models for the WHOQOL-BREF domains with Gestational Age as a Categorical Variable

| Variable | Domain 1  (Physical health),  n = 3021 | *p* value | Domain 2 (Psychological),  n =3020 | *p* value | Domain 3  (Social Relationship),  n = 2973 | *p* value | Domain 4 (Environment),  n = 3020 | *p* value |
| --- | --- | --- | --- | --- | --- | --- | --- | --- |
| Gestational Categories |  |  |  |  |  |  |  |  |
| Full-Term (ref) |  |  |  |  |  |  |  |  |
| Preterm | 0.099 (-0.145-0.344) | 0.425 | -0.009 (-0.224- 0.243) | 0.938 | -0.005 (-0.273-0.263) | 0.971 | -0.010 (-0.292-0.272) | 0.944 |
| Nationality |  |  |  |  |  |  |  |  |
| Malaysian (ref) |  |  |  |  |  |  |  |  |
| Non-Malaysian | -0.290 (-0.583 –  0.003) | 0.053 | -0.285 (-0.550-  -0.019) | 0.035* | -0.204 (-0.490-  0.082) | 0.163 | -0.243 (-0.595- 0.110) | 0.177 |
| Ethnicity |  |  |  |  |  |  |  |  |
| Malay (ref) |  |  |  |  |  |  |  |  |
| Chinese | 0.067 (-0.123-0.256) | 0.490 | - | - | -0.262 (-0.469-  -0.056) | 0.013* | 0.059 (-0.162-0.281) | 0.599 |
| Indian | -0.192 (-0.424-0.040) | 0.105 | - | - | 0.006 (-0.276-0.288) | 0.967 | 0.378 (0.093-0.663) | 0.009* |
| Other Bumiputera | 0.894 (0.595-1.193) | <0.001** | - | - | -0.295 (-0.577-  -0.013) | 0.040* | -0.968 (-1.298-  -0.637) | <0.001** |
| Others | 0.201 (-0.338-0.739) | 0.465 | - | - | -0.261 (-0.757-  0.235) | 0.303 | -0.240 (-0.800-  0.320) | 0.400 |
| Highest Education Level |  |  |  |  |  |  |  |  |
| University (ref) |  |  |  |  |  |  |  |  |
| Never attended school, Primary | -0.423 (-0.721-  -0.126) | 0.005* | -0.583 (-0.842-  -0.324) | <0.001** | -0.300 (-0.602-  0.002) | 0.051 | -1.217 (-1.527-  -0.907) | <0.001** |
| Secondary | -0.210 (-0.414-  -0.006) | 0.043* | -0.428 (-0.605-  -0.250) | <0.001** | -0.263 (-0.462-  -0.064) | 0.010* | -0.705 (-0.891-  -0.518) | <0.001** |
| College (Pre-University) | -0.052  (-0.306-0.202) | 0.688 | -0.296 (-0.521-  -0.071) | 0.010* | 0.041 (-0.203-  0.287) | 0.740 | -0.195 (-0.429-  0.039) | 0.103 |
| Marital Status |  |  |  |  |  |  |  |  |
| Married (ref) |  |  |  |  |  |  |  |  |
| Single, Separated/Living Apart, Divorced, Widowed | - | - | -0.167 (-0.766-  0.431) | 0.583 | -0.225 (-0.830-  0.380) | 0.466 | -0.105 (-0.652-  0.442) | 0.706 |
| Primary support |  |  |  |  |  |  |  |  |
| Husband (ref) |  |  |  |  |  |  |  |  |
| Parents | - | - | -0.438 (-0.763-  -0.113) | 0.008* | -0.374 (-0.754-  0.006) | 0.054 | -0.183 (-0.553-  0.186) | 0.331 |
| Mother-in-law | - | - | -0.796 (-1.582-  -0.011) | 0.047* | -1.348 (-2.342-  -0.354) | 0.008* | -0.559 (-1.299-0.181) | 0.139 |
| Extended family/Friend, Paid help, Others | - | - | -0.857 (-1.807-  0.093) | 0.077 | -0.728 (-1.849-  0.394) | 0.203 | -0.706 (-1.578-  0.166) | 0.112 |
| Sex of infant |  |  |  |  |  |  |  |  |
| Male (ref) |  |  |  |  |  |  |  |  |
| Female | - | - | - | - | -0.114 (-0.244-0.017) | 0.087 | - | - |
| Infant’s birth weight (kg) | - | - | - | - | - | - | 0.001 (-0.152-0.155) | 0.986 |
| Type of delivery |  |  |  |  |  |  |  |  |
| Standard Vaginal/ Breech delivery (ref) |  |  |  |  |  |  |  |  |
| Forceps/Vacuum vaginal delivery | 0.246 (-0.186-  0.678) | 0.264 | -0.034 (-0.431-  0.363) | 0.868 | -0.017 (-0.464-  0.429) | 0.940 | 0.032 (-0.398-  0.462) | 0.884 |
| Elective Caesarean | 0.029 (-0.228-0.287) | 0.823 | 0.037 (-0.301- 0.227) | 0.784 | -0.098 (-0.380-0.183) | 0.494 | 0.193 (-0.077-0.463) | 0.161 |
| Emergency Caesarean | -0.279 (-0.469-  -0.089) | 0.004* | -0.322 (-0.507-  -0.138) | 0.001** | -0.201 (-0.400-  -0.002) | 0.048 | -0.337 (-0.530-  -0.143) | 0.001** |
| APGAR score (1 min) | 0.101 (0.046-0.156) | <0.001* | 0.058 (0.007-0.110) | 0.027* | - | - | 0.017 (-0.040-0.074) | 0.568 |

Values presented are the unstandardised beta coefficients (B) and corresponding 95% CIs;

^a^All mothers answered ‘Yes’ for this variable;

* Statistically significant (*p*≤0.05); ** Statistically significant (*p* ≤0.001).
